# Functional Living Skills: A Non-Immersive Virtual Reality Training for Individuals with Major Neurocognitive Disorders

**DOI:** 10.3390/s21175751

**Published:** 2021-08-26

**Authors:** Simonetta Panerai, Donatella Gelardi, Valentina Catania, Francesco Rundo, Domenica Tasca, Sabrina Musso, Giuseppina Prestianni, Stefano Muratore, Claudio Babiloni, Raffaele Ferri

**Affiliations:** 1 Unit of Psychology I.C., Oasi Research Institute-IRCCS, 94018 Troina, Italy; spanerai@oasi.en.it (S.P.); dgelardi@oasi.en.it (D.G.); vacatania@oasi.en.it (V.C.); dtasca@oasi.en.it (D.T.); smusso@oasi.en.it (S.M.); gprestianni@oasi.en.it (G.P.); smuratore@oasi.en.it (S.M.); 2 Unit of Bioinformatics and Statistics, Oasi Research Institute-IRCCS, 94018 Troina, Italy; frundo@oasi.en.it; 3 Department of Physiology and Pharmacology “V. Erspamer”, Sapienza University of Rome, 00185 Rome, Italy; claudio.babiloni@uniroma1.it; 4 Unit of Neurology I.C., Oasi Research Institute-IRCCS, 94018 Troina, Italy

**Keywords:** virtual reality, neurocognitive disorder, dementia, functional living skills

## Abstract

The loss of functional living skills (FLS) is an essential feature of major neurocognitive disorders (M-NCD); virtual reality training (VRT) offers many possibilities for improving FLS in people with M-NCD. The aim of our study was to verify the effectiveness of a non-immersive VRT on FLS for patients with M-NCD. VRT was carried out for 10 to 20 sessions, by means of four 3D apps developed in our institute and installed on a large touch screen. The experimental group (EG) and the control group (CG) included 24 and 18 patients with M-NCD, respectively. They were administered the in vivo test (in specific hospital places reproducing the natural environments) at T1 (pre-training) and T3 (post-training); at T2, only EG was administered VRT. Statistically significant differences between EG and CG in all the in vivo tests were found in the number of correct responses; during VRT, the number of correct responses increased, while the execution times and the number of clues decreased. The improvement in the in vivo tests appeared to be related to the specific VRT applied. The satisfaction of participants with the VRT was moderate to high.

## 1. Introduction

Cognitive deficits, representing a decline from a previous level of functioning, are the core symptoms of major neurocognitive disorder (M-NCD) [1], or dementia. The cognitive impairments interfere with the independence in the functional living skills (FLS) of patients who then need aids from others for carrying out activities that they were previously able to complete on their own. FLS can be defined as the activities that people have to perform daily in order to be integrated in their community, to live independently, and exercise their own role; FLS include complex skills such as driving and mobility, financial management, meal preparation and cleanup, and shopping [2]. When these skills deteriorate, patients lose their self-sufficiency and, consequently, their self-esteem and well-being [2,3]. FLS impairments are also related to an augmented risk of caregiver burden and institutionalization [4]. It is estimated that 35.6 million people currently live with dementia in the world, and this number should reach 115.4 million people by 2050 [5]. Treating dementia symptoms therefore represents a challenge for clinicians and the scientific community, and pharmacological treatments fail to sufficiently control or decrease them [6]. Therefore, based on the theoretical foundation of both neuronal plasticity and cognitive reserve [3], cognitive interventions have been increasingly focused as an emerging therapeutic approach to enhance quality-of-life, improve or maintain cognitive and functional living skills, and improve psychological and behavioral symptoms of dementia [7]. Currently, cognitive interventions for people with NCDs can count on new and very promising technological approaches, such as virtual reality (VR) [8], which seems to be more effective than traditional approaches, producing positive outcomes in a shorter time, as well as enhancing the brain neuroplasticity [9]. VR provides three-dimensional life-like environments at a full (e.g., virtual camera or oculus) or non-immersive level (e.g., the desktop visual display system), allowing patients to do an interactive experience, based on “learning by doing” (which integrates two different cognitive systems, intuition and reasoning) [10,11].

While immersion and sense of presence are considered to belong to a continuous spectrum, VR systems are frequently divided into non-immersive and immersive in relation to their immersiveness (i.e., capability to immerse the user) [12,13,14]. Non-immersive VR refers to virtual environments projected onto two-dimensional displays, such as computer screens, laptops, tablets, and mobile devices [12,13,14]. However, non-immersive VR tests can be challenging for individuals without gaming backgrounds [15], especially for older adults and clinical populations (e.g., individuals with mild cognitive impairment or Alzheimer’s disease) [16,17]. In contrast, immersive VR refers to virtual environments that are projected onto devices such as head-mounted displays (HMDs) and CAVE systems [12,13,14,18]. Non-immersive VR provides a less ecologically valid testing environment than immersive VR tests [19,20,21,22]. Importantly, differences in performance between gamers and non-gamers are mitigated by the first-person view and ergonomic interactions that are proximal to real-life actions in immersive VR environments [15]. Consequently, an immersive VR research paradigm might provide the most efficient approach to study everyday performance.

Studies in the literature are predominantly focused on the diagnostic assessment of dementia. In this regard a recent article [23] validated the Virtual Reality Everyday Assessment Lab (VR-EAL), an immersive VR neuropsychological battery for evaluating cognitive everyday functions, vs. an extensive paper-and-pencil neuropsychological battery. This study is relevant to ours because it seems to present some similarities; indeed, it examined the ecological validity, pleasantness-satisfaction, computer/gaming competency, and the evaluation of everyday performance. Correlations between the VR-EAL scores and their equivalent scores on the paper-and-pencil tests were statistically significant; furthermore, the VR-EAL required less administration time and the participants judged it more ecologically valid and pleasant than the traditional neuropsychological battery.

Studies examining the effectiveness of VR interventions are limited [24]. Recent reviews on the use of VR with people with NCDs [24,25] found small-to-medium effects with greater effect sizes when using semi-immersive than full-immersive technologies; improvements involve motor and cognitive skills (such as memory, attention, but also coping strategies) and psychological symptoms (such as anxiety). As far as FLS are concerned, the results of earlier studies suggest that VR training (VRT) might improve the performance of patients with dementia and produce outcomes similar to those of training carried out in the natural environment [25,26,27,28,29,30]. The study by Hofmann et al. [26] showed improvements in a shopping task, with decreased errors and times; a follow-up after three weeks showed that people maintained their skills; the participants to the study also appreciated the proposed interactive modality. Van Schaik et al. [28] investigated both facilitators (e.g., directional signals) and barriers (e.g., motor traffic surface) for a walk in a city center, providing suggestions for successful types of landmarks; the authors compared the performance in the real world, within VR reproducing real world, and in adapted VR. They found a performance improvement in the walk-task, especially in the adapted VR model. On the contrary, in the VR tasks participants showed a less safe behavior (e.g., when crossing a road). Yamaguchi et al. [25] proposed a dual-modal VR kitchen in order to re-learn two simple cooking tasks (preparing a toast and a cup of coffee); written or vocal instruction were provided in order to prevent errors. The study provided preliminary support for a VR therapy based on the dual-modal procedure in patients with Alzheimer’s disease, who increased their accomplishment scores and decreased perseverations and the use of verbal and written additions. Manera et al. [29] examined the acceptability of the “kitchen and cooking” game installed on a tablet, and confirmed the utility of its use for rehabilitation purposes; patients improved their performances in one week, they were faster in both executive and praxis functions. This game seemed to also work for patients in whom dementia was associated with apathy. Foloppe et al. [30] used VRT with a patient with Alzheimer’s disease to re-learn four cooking activities, suggesting that it can produce improvements in the same way as the real-life relearning. Finally, Fasilis et al. [31] evaluated the effectiveness of an interactive computer training on three FLS (shopping from the supermarket, preparing a breakfast, and tide up and clean the house), with three possible levels of increasing difficulty for each task. After training, increased scores in some memory (story recall) and general executive functions were found; on the contrary, no improvements were found in working memory, attention and problem solving.

Some suggestions might be derived from the studies described above, focused on the rehabilitation or re-learning of some FLS: (a) each training lasted for at least four weeks; (b) before beginning specific training, familiarization training was conducted; (c) games included some aids (verbal, written, or video demonstrations) to facilitate the correct responses; (d) outcomes were measured by means of neuropsychological testing [26,31], or training variable scores (such as time spent for completing the tasks, latency time, number of errors, correct actions, number of aids) [25,26,28,29,30]; one study [30] used self-esteem, quality of life, and caregiver burden measures; (e) some studies included a non-structured acceptability/agreement questionnaire [26,29] in order to obtain information on the personal experience of each participant; (f) two studies [28,30] also carried out a comparison between VR and real world performance, in order to obtain information on the VRT effectiveness compared with traditional teaching in the natural environment.

With the study reported here, we focused on patients with M-NCD and aimed to: (a) verify if patients might be successfully trained in four specific FLS (to provide information, to take medicines, to prepare a suitcase, and to shop at the supermarket) by using a non-immersive VRT including four apps installed on a touch-TV; and (b) verify if transfer from virtual to real environment occurred, by administering in vivo tests before (T1) and after (T3) the VRT. We selected a non-immersive environment because it allows an easier continuation of rehabilitation at home (which is an important objective of our team), in comparison to the immersive reality systems. Furthermore, the non-immersive VR limits the risk of simulation sickness, a problem described in elderly people during virtual assessment [32], including symptoms such as discomfort, apathy, drowsiness, disorientation, fatigue, and nausea. Finally, the non-immersive VR allows patients with dementia to maintain the temporo-spatial coordinates of the environment in which the training occurs; therefore, he/she feels less disoriented and more able to maintain a control on the environment, compared to an experience of immediate transfer into a completely new reality.

A previous study by our team verified the feasibility of such a VRT [33].

## 2. Materials and Methods

### 2.1. Participants

Participants were recruited at the Unit of Neurorehabilitation, Brain Aging Department, Oasi Research Institute-IRCCS, Troina, Italy during the years 2017–2020. Inclusion criteria were: (a) diagnosis of M-NCD, according to the DSM-5 criteria [1]; (b) score from 10 to 24 at the Mini-Mental State Examination (MMSE) [34]; (c) loss of one or more instrumental activities of daily living (IADL) [35]; (d) sufficient sight, hearing, and motor functioning; (e) sufficient communication skills; (f) maintained reading skills. Exclusion criteria were: (a) diagnosis of mild-NCD [1] or mild cognitive impairment; (b) severe dementia; (c) aphasia; (d) severe sight, hearing, or motor impairments; (e) loss of reading skills. Patients were assigned to the experimental group (EG) if they agreed to the VRT (*n* = 24); patients who did not accept VRT but were available for the two in vivo assessments, were assigned to the control group (CG; *n* = 18).

### 2.2. System and App Description

The system is based on a client-server architecture through the use of REST API. The server consists of a web service built in PHP and a database designed according to the project needs and built using the PostgreSQL engine. The communication between the clients (the apps installed on touch TV) and the database takes place through REST calls. Specific URLs allow both to read data stored in the database and to write parameters of output coming from the applications running on the devices. The exchange of data between the service and the apps takes place via the JSON format (JavaScript Object Notation), a standard for exchanging data on the web. The system is equipped with an application developed in Visual Studio that allows to set the parameters of the app to be run on the devices; furthermore, it allows the management of the data stored in the database. The apps have been developed using the Unity 3D multiplatform graphics engine (C# language), so as to be usable on different types of clients.

Four apps were developed, for some areas of instrumental activities of daily living identified by the American Association of Occupational Therapy: communication, health, home management, and shopping [2]. Different levels of difficulty characterize each app: three include a single three-dimensional scene, one runs through three different scenes; in two of the apps the patient is requested to provide responses by touching one item of the scene; dragging an object from one place to another is required in two apps.

#### 2.2.1. App Information

The subject is required to provide information by answering 30 questions appearing on the screen in verbal and written form, with multiple-choice answers; questions concern general knowledge, personal, family, spatial, and temporal orientation.

#### 2.2.2. App Medicines

The subject is required to indicate the correct time of the day to take medicines. Five medicine boxes are presented placed on a kitchen table, verbal instructions, as well as a visual reminder indicate when each drug should be taken; subsequently, the patient is required to touch one of the medicine boxes in the scene as a response to 10 verbal requests, randomly presented during each session; the schedule of the daily treatment is always available on the screen.

#### 2.2.3. App Suitcase

The subject is required to fill up a suitcase for a weekend out. A single scene is used, with clothes on shelves that need to be placed in a suitcase by dragging them, following verbal instructions.

#### 2.2.4. App Supermarket

The subject is requested to shop at the supermarket with a shopping list, which includes five items and is available on the screen on demand. A kitchen is the scenario of the first scene, in which the shopping list, money, and wallet must be taken. The second scene shows a supermarket shelf with different items. Items included in the shopping list must be dragged from the shelf and put into a shopping cart. The third scene includes the cash counter; items need to be dragged from the cart onto the cash counter and paid for by dragging some notes from the wallet.

The apps were arranged following the principles of applied behavior analysis (ABA) within the behavioral psychology field, an evidence-based best practice, including principles and procedures used for planning several different behavioral treatments [36]. The joint use of ABA and VR appears to be promising [37]. In our study, several ABA procedures were introduced, such as verbal reinforcement after a correct response (e.g., “good!”, “ok!”, “congratulations!”, “well done!”), a least-to-most prompting (up to a maximum of three clues) after errors in responses, task analysis, and total task chaining for two apps (supermarket and suitcase). A video demonstration before starting the task is available for the supermarket and suitcase apps. For each app, an explanation for how to perform the task is provided at the beginning, simultaneously, in a verbal and written form.

A detailed description of the above apps is reported in the Supplementary Materials.

### 2.3. Procedures

This work is a nonrandomized interventional comparison study, including three assessments at times T1, T2, and T3, as detailed below:

#### 2.3.1. T1—Pre-Treatment Assessment for Both EC and CG

Upon admission for treatment of cognitive intervention, all patients were administered a neuropsychological battery (as an usual part of the admission process) by a clinical psychologist, blinded to the objectives of the study; the battery included one test for assessing reasoning ability and intellectual level, the colored progressives matrices [38]; two span test, the Visuo-spatial test of Corsi and the Digit span [39]; one test for assessing the immediate and delayed verbal episodic memory, Rey’s 15 words [40]; one test for assessing the frontal functioning, the Frontal Assessment Battery [41]. Two scales for assessing the activities of daily living were also administered: the Katz index of independence in activities of daily living (ADL) [42] for basic activities, such as bathing, toileting, dressing, and eating; and the IADL [35] for daily activities at home and in the community that require more complex interactions than the ADL. The first in vivo tests were administered into real environments arranged in our institute by a different clinical psychologist, specialized in cognitive-behavioral psychology, unaware of whether the patient was enrolled in the EG or CG. During the administration, no clues or reinforcements were provided; both the number of correct responses and the total execution time were recorded. Participants were also administered a digital literacy questionnaire, which showed that 58% of them were able to use only one technological device (cellular phone) and 25% two devices (cellular phone and tablet). Table 1 shows the scores obtained at neuropsychological testing, as well as the scores at the in vivo tests, for both EG and CG, and the results of the comparisons between the two groups.

#### 2.3.2. T2: Treatment

All participant benefited from the group intensive cognitive activation (g-ICA) intervention [3] carried out in our Unit of Psychology I.C. g-ICA is an intensive combined group treatment, combining cognitive stimulation and cognitive training that includes 30 cognitive activation sessions delivered by a trained clinical neuropsychologist, supported by a practicing psychologist. Each group includes four to five participants. Daily group sessions are scheduled, each lasting approximately 3 h, for 5 days a week (from Monday to Friday), over a period of 2 months. Cognitive contents are organized on a weekly basis with the aim to stimulate a wide range of cognitive functions (global cognitive function; ecological memory; ideomotor and constructional praxis; visual—auditory—and spatial memory; selective attention; verbal language; etc.); the level of difficulty of each activity is adapted to group capability. Both paper-pencil and computer tasks are employed to train specific cognitive functions.

During T2, EG was also administered the VRT for 10 to 20 five-day-per-week sessions, based on the duration of the patient’s hospitalization period. All the EG participants were administered 10 sessions, 22 participants 12 sessions, 20 participants 13 sessions, 19 participants 15 sessions, 15 participants 16 sessions, 14 participants 17 sessions, and 12 participants 20 sessions. Before starting VRT, two to three trial app sessions were administered to become familiar with the technological tool, and to learn the dragging movement. The patients performed the VRT tasks in the presence of a psychologist, who intervened only in the case of system malfunctioning. The patients were required to autonomously manage the tasks and the device. The following parameters were recorded: total execution time, number of correct responses (for the supermarket and suitcase tasks, the number of correct task steps), number of errors, number of missing responses (participants not answering within 10 s), number of clues provided to obtain a response.

#### 2.3.3. T3: Post Treatment Assessment for Both EG and CG

The post treatment assessment included a second administration of both neuropsychological battery and in vivo test for both patient groups and the administration of a satisfaction questionnaire to the EG, specifically developed by the research team. It included 14 questions with three response options: 0 = low, 1 = moderate, 2 = high. Part I (first eight questions, maximum total score 16) focused on the system use (easy/hard), the actual possibility of learning throughout the VRT, the level of personal satisfaction, and the desire to continue the experience. A total score ranging from 0 to 4 and from 13 to 16 were assumed to be indicative of low and high satisfaction level, respectively; scores from 5 to 8 and from 9 to 12 were considered as low-moderate and moderate-high, respectively. Part II (last six questions, maximum total score 12) focused on the technological problems encountered and negative feelings and symptoms (e.g., boredom, fatigue, anger, mental confusion, etc.). Scores from 0 to 3 were assumed to be indicative of a low level of problems, from 4 to 6 low-moderate, from 7 to 9 moderate-high, and from 10 to 12 high. The satisfaction questionnaire is shown in the Supplementary Materials.

### 2.4. Statistical Analysis

Asymmetry and kurtosis calculations failed to show a normal distribution of the data recorded, so nonparametric statistics were used. Between-group in vivo test comparisons were done with the Mann Whitney U test, and r effect sizes were calculated (r = z/√N). The r value of 0.1, 0.3, and 0.5 indicate a small, medium, and large effect size, respectively. The Chi square test was used to analyze between-group differences in dementia etiology and severity or gender. A within-group analysis was also added by using the Wilcoxon matched pairs test. For within- and between-group comparisons, significance was set at *p* < 0.05. After the Wilcoxon matched pairs test, the Bonferroni correction was applied, setting the *p* value at 0.0025. The Friedman test for repeated measures was used for the VRT data; effect sizes were calculated by means of the Kendall’s W coefficient of concordance, which uses the Cohen’s interpretation guidelines of 0.1 = small effect, 0.3 = moderate effect, and 0.5 = strong effect [43]. For each parameter (correct responses, errors, missing responses, number of clues, and average response time) comparisons were carried out by means of the Wilcoxon matched pairs test, between the first and each of the subsequent executions of the tasks. A second analysis was also added to know in which specific sessions a statistically significant change occurred; so, we carried out comparisons between the first execution and the following ones, until finding the session in which a statistically significant difference occurred; then, between this last one and the following ones, and so on, up to the 20th session.

Finally, the Spearman rho correlation coefficient was used to analyze the correlation between IADL and performance at the VRT.

## 3. Results

As shown in Table 1, at T1 no significant differences between EG and CG were found, neither in dementia etiology or severity, gender, chronological age, and years of education, nor in the neuropsychological battery scores. Moreover, for the first in vivo test, no statistically significant differences were found neither in total execution time nor in correct responses, except for the total execution time for information and medicines (the CG took longer to conclude the tasks). Therefore, the two groups could be considered comparable.

As far as the comparisons between the neuropsychological batteries at T1 and T3 are concerned, statistically significant differences (Wilcoxon matched pairs test) were found in the EG group involving MMSE (z = 3.17, *p* = 0.0015, r = 0.65), immediate recall of the Rey’s 15 words (z = 2.98, *p* = 0.003, r = 0.61), and the Frontal Assessment Battery (z = 2.1, *p* = 0.03, r = 0.43); in the CG group, statistically significant differences were found for MMSE (z = 2.57, *p* = 0.01, r = 0.61), colored progressive matrices (z = 3.24, *p* = 0.0012, r = 0.76), delayed recall of the Rey’s 15 words (z = 2.2, *p* = 0.03, r = 0.52), and Frontal Assessment Battery (z = 2.1, *p* = 0.04, r = 0.49). No statistically significant differences were found when comparing the differences in neuropsychological batteries at T3 and T1 between the EG and the CG groups.

Table 2 shows statistically significant differences found in the comparison between the second (T3) and the first (T1) in vivo tests. For all the apps, the number of correct responses significantly increased in the EG, as well as the total execution time for information and medicines. On the contrary, no statistically significant differences were found in the within-group analysis for the CG. In the EG, the number of patients who reached a percentage ≥ 80% of correct responses, increased in all the tasks between T1 and T3, and these differences were statistically significant for three out of the four apps (suitcase and supermarket: *p* < 0.05; medicine: *p* < 0.01; Chi square test).

The between-groups analysis showed statistically significant differences in number of correct responses, with large (suitcase and medicines) to moderate (information and supermarket) effect sizes. No differences were found in the total execution time, except for suitcase, with a medium effect size (CG participants appeared to be faster than EG participants). During the VRT sessions (Table 3) administered to EG, statistically significant differences were found for all the apps in number of correct responses and missing responses (with small to moderate effect sizes), as well as in number of clues (with small effect sizes) and in total execution times (with moderate to strong effect sizes). No statistically significant differences were found in number of errors.

**Figure 1:**
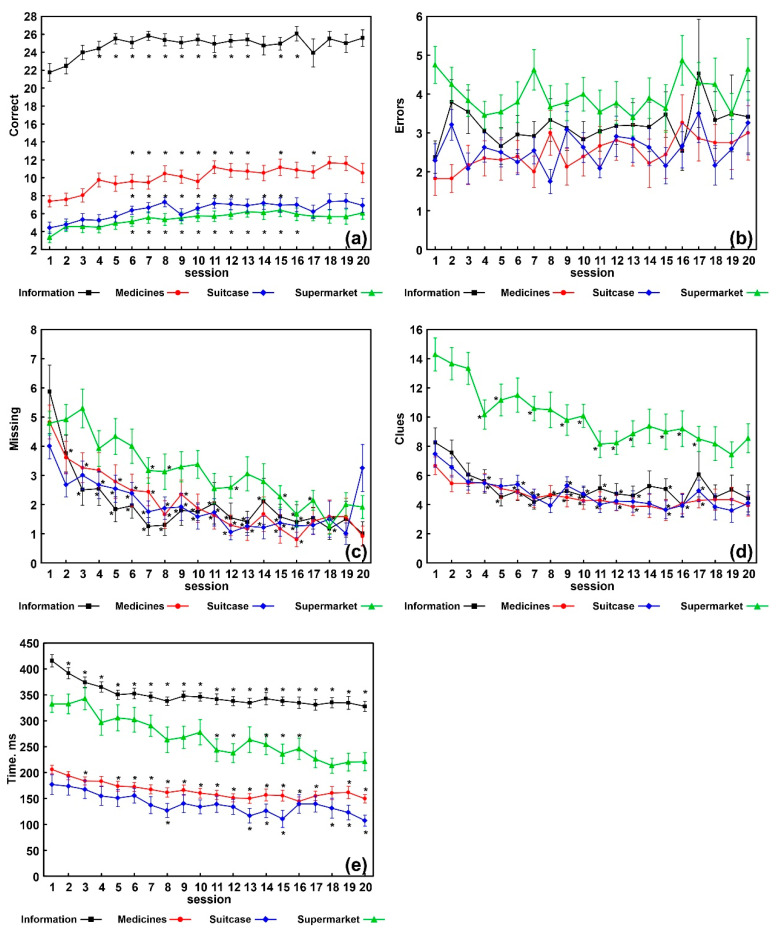
Results obtained in each of the 20 sessions in each task; data are shown as mean (squares, circles, diamonds, and triangles) and standard errors (whiskers), asterisks indicate a statistically significant difference (Wilcoxon rank test, Bonferroni-corrected *p* < 0.05 vs. the value obtained for session 1. (**a**) Number of correct responses; (**b**) number of errors; (**c**) number of missing responses; (**d**) number of clues provided; (**e**) average execution time during the task.

Figure 1 graphically shows the trend over time of all variables evaluated during the 20 VRT sessions and includes the statistically significant differences between the first session and the subsequent ones. In the correct responses, differences remained statistically significant from session 5 to session 15–17, in all the apps.

In the missing responses differences remained statistically significant from session 2–4 to session 16–18 in information, medicines, and suitcase apps; from session 7 to session 18 for the app supermarket. In the number of clues, from session 3–4 to session 16–17, in all the apps. For execution time, from session 2–3 to session 20 for information and medicines; from session 8 to session 20 for suitcase; from session 11 to session 16, for supermarket. No statistically significant differences were found for number of errors.

The results of the analysis carried out in order to know in which specific sessions a statistically significant change occurred, can be summarized as follows:

- Information showed 3-to-5 improvement steps (with respect to the preceding session) in correct responses and clues at sessions 3, 5, and 20 t, *p* = 0.004, *p* = 0.003, and *p* = 0.05, respectively; missing responses at sessions 2, 3, and 7, *p* = 0.014, *p* = 0.009, and *p* = 0.004, respectively; execution time, at sessions 2, 3, 5, 12, and 19, *p* = 0.0007, *p* = 0.03, *p* = 0.0007, *p* = 0.01, and *p* = 0.05, respectively;
- Suitcase showed 2-to-4 improvement steps for each variable (correct responses at sessions 3 and 6, *p* = 0.04, respectively; clues at sessions 3 and 7, *p* = 0.002 and *p* = 0.01, respectively; missing responses at sessions 2, 7, and 12, *p* = 0.005, *p* = 0.005, and *p* = 0.01, respectively; total time execution at sessions 4, 7, 12, and 20, *p* = 0.001, *p* = 0.0005, *p* = 0.005, and *p* = 0.03, respectively);
- Medicine showed 2-to-3 improvement steps for each variable (correct responses and clues at sessions 2 and 7 t, *p* = 0.009 and *p* = 0.04, respectively; missing responses at sessions 2, 6, and 13, *p* = 0.03 for all; execution time at sessions 3, 7, 11, and 20, *p* = 0.00007, *p* = 0.014, *p* = 0.03, and *p* = 0.05, respectively);
- Supermarket showed 2-to-3 improvement steps for each variable (correct responses at sessions 4 and 15, *p* = 0.003 and *p* = 0.03, respectively; clues at sessions 4 and 19, *p* = 0.0005 and *p* = 0.03, respectively; missing responses at sessions 7 and 16, *p* = 0.0009 and *p* = 0.0015, respectively; execution time, at sessions 6, 11, and 16, *p* = 0.04, *p* = 0.0003, *p* = 0.001, respectively).

The analysis of the correlation between baseline IADL values and performance at the VRT showed that scores on IADLs negatively correlated with the number of errors in medicine (rho = −0.49, *p* < 0.01) and suitcase (rho = −0.44, *p* < 0.025); the scores on the Frontal Assessment Battery correlated negatively with the number of aids in the suitcase (rho = −0.49, *p* < 0.01) and in the supermarket (rho = −0.46, *p* < 0.025); MMSE scores correlated with missing supermarket responses (rho = 0.41, *p* < 0.025); the scores at Rey’s 15 words, deferred memory, correlated with the number of aids at the supermarket (rho = 0.46, *p* < 0.025).

A detailed table of the patient individual data is also included into the Supplementary Materials.

Finally, the median score obtained in part I of the satisfaction questionnaire was 11 (interquartile range: 8–13); in part II it was 0 (interquartile range: 0–1).

## 4. Discussion

Loss of functioning in complex tasks of everyday life is a hallmark feature of M-NCD and constitutes the second diagnostic criterion for dementia [1]. FLS are complex skills involving motor, cognitive, and social abilities, and their impairments can present in both early dementia and mild cognitive impairment [44]. They can be understood as multitasking skills, closely related to executive functioning and prospective memory. They are also related to self-perception and self-esteem and affect personal and family well-being. Treatments to maintain or slow down the decline of FLS might reduce hospital admissions, use of emergency services, and caregiver overload [2].

VRT is acquiring increasing credibility as a useful tool for reinforcing FLS of persons with cognitive disorders; studies on this topic, although still few, are very promising. Our study aimed to verify the effectiveness of a non-immersive VRT on FLS of patients with M-NCD, as well as its ecological value if the re-learned skills are then shown to be spontaneously (without further teaching) transferred to the natural environments. In our work, we tried to overcome some limitations of previous studies, taking into account some of the recommendations by Moreno et al. [27]. Therefore, we provided clinical information on the sample, assessed user acceptance and eventual adverse effects, reported effect sizes for the outcomes, and provided example of generalization to the real environment by means of the in vivo tests.

The results of our study refer to: the effectiveness of VRT on the re-learning of four FLS (information, medicine, suitcase, and supermarket); how VRT implemented in our institute works; the spontaneous transfer to the natural environment of the re-learned FLS; the patient satisfaction with non-immersive VRT.

As far as the effectiveness of VRT is concerned (Table 3), we found increased correct responses, as well as a decrease in missing responses, number of clues, and execution times. We obtained *p* values much lower than 0.05 in almost all the variables taken into account for all the apps (except for errors); therefore, our non-immersive VRT seems to have induced a strong change in our participants with M-NCD. However, the effect sizes were low to moderate for both correct and missing responses; these results are likely to be related to the individual variability within our EG. This individual variability indicates the need to assess, in future studies, the personal and clinical features possibly linked to the achievement of the best outcomes. On the contrary, the effect sizes of the parameter execution time were moderate to high; therefore, in this case, our results might be considered more homogeneous. No statistically significant differences were found in number of errors, whose average values (from 2 to 5) in all the tasks are shown in Figure 1b. It seems that our subjects made omissions (Figure 1c) rather than errors, especially in the first sessions of the VRT, which could be linked to the hesitation characterizing people with dementia. Over the sessions, the omissions decreased significantly, so we can hypothesize that the patients gained confidence in carrying out the tasks. On the contrary, errors remained almost stable. These last data could probably be related to the typical difficulties in learning new tasks in patients with dementia. Indeed, VRT, while providing very familiar tasks, represented a new learning system with which patients had never faced up to that moment.

How does VRT implemented in this study work? The comparisons carried out between the first and the following sessions showed significant changes, starting within the first five sessions, up to sessions 16–18 and, in the case of the execution time, up to session 20 (Figure 1). Furthermore, as described above in the results section, for each variable of each app, two to three statistically significant steps of improvement were found. The most significant changes occurred within the first 10 sessions (especially within the first seven); in the subsequent sessions (11 to 20) some improvements could still be found, but to a much lesser extent. Our opinion is that these findings might be relevant, as they might guide decision on the best VRT administration format, to obtain the best outcomes as well as to prevent boredom and fatigue (for example, 20 consecutive sessions? or two or more cycles of 10 sessions each, interspersed with breaks?). Therefore, these findings need to be further confirmed with larger samples, since in our EG only 12 patients were administered 20 VRT sessions.

Regarding the spontaneous transfer to the natural environment of the re-learned FLS, the in vivo tests (Table 2) showed only in EG statistically significant differences between T3 and T1, especially in the number of correct responses. Moreover, in the between-groups analysis, statistically significant differences in number of correct responses were found, with moderate-to-large effect size. Since both groups participated in a group intensive cognitive activation treatment, these findings suggested that only cognitive stimulation is not enough to re-learn FLS, but that FLS need a specific training to be re-learned. Indeed, both groups showed statistically significant differences with large effect sizes in some neuropsychological tests assessing either general cognitive functioning, or verbal memory, or executive functioning; no differences were found when comparing the two groups. We might hypothesize that the improvements obtained by both EG and CG in the neuropsychological tests are due to the g-ICA treatment from which both groups benefited, while only the EG group, which benefited from the VRT, showed such improvements in the in vivo tests at T3 as to produce a statistically significant difference in comparison to CG. The absence of improvement in the IADL scale scores in both groups might be explained by the characteristics of the scale. The IADL scale assesses a total of eight domains, evaluating a specific skill for each domain (e.g., using a telephone, doing laundry, handling finances, and so on). It can detect any changes occurring in those specific skills. A limitation of the IADL scale is that it may not be sensitive to small, incremental changes in function [45]. These findings also show that a spontaneous transfer of the re-learned FLS to the natural living environments is achievable, thus suggesting the ecological validity of the non-immersive VRT. As said in the Introduction, VRT provides an interactive experience based on “learning by doing”. Learning by doing requires the activation of the intuitive cognitive system, which generates impression of the attributes of perceived objects/situation in a rapid and non-voluntary way. These characteristics can be recognized also in other conditions, spontaneously generating a transfer of the behavior from a situation to another.

Concerning the satisfaction questionnaires, scores were indicative of a moderate-high satisfaction; furthermore, little or no-problem in using the device, as well as no adverse events were reported by the participants. Among the technical problems, patients mentioned only a sudden termination of the apps which, however, occurred sporadically. Therefore, our results confirm data from the literature reporting that VR seems to be generally accepted and considered interesting and motivating by people with NCDs, with only minor adverse effects [27]. However, some difficulties reported by the participants in performing the tasks might be taken into consideration in order to improve the development of new apps: for example, the blinking highlighting of some items was not sufficiently marked; six alternatives for one response each question in the information app were considered too many by some patients; in the medicine app three verbal requests were slightly different from the visual reminder and required an inference, a difficult mental process for some of our patients (e.g., the visual reminder showed to take the painkiller after the afternoon snack, while the question was “It’s 6:00 p.m., what medicine do you take?”); finally, the reminders which were available after clicking on a button, required the participants to remember to click, but unfortunately some of our patients did not have such a memory ability.

Our study has some limitations: the sample size is small; on the other hand, the effect sizes are moderate to large, which at least partially mitigate the small sample size of the study. Patients with M-NCD due to different etiologies were included, this did not allow us to know if different clinical groups might engage in VRT differently and reach different outcomes. The number of VRT sessions administered to the patients was variable, and only few patients were administered 20 sessions; future studies with larger samples might better investigate the effects of a longer VRT on the re-learning of FLS. The tests in vivo were administered in environments arranged in our Institute; therefore, we did not collect data on patient behavior in their own home. The VRT tasks were the same of the in vivo test, so that one might conclude that the outcomes are only due to practice. VRT is based on a specific learning approach, the learning by doing, that is the process of acquiring skills by practical activities and personal experiences. This explains the better performance of patients in the four VR tasks. Conversely, the doing per se does not sufficiently explain the better performance on the in vivo test. In this case, the generalization process (in the sense proper to the behavioral psychology) most likely also occurred, for spontaneously transferring the skills learned in the training environments to the natural contexts, after training ended. The generalization process is related to the integrity of cognitive and hippocampal functioning [46,47]. In people with dementia, due to the cognitive decline, the generalization process can be impaired. The results of our study seem to suggest that VRT might promote, thanks to the activation of the intuitive system (as said above), the skills generalization from one context to another, at least in the early stages of dementia. Finally, our study did not include a follow-up, therefore we had no knowledge of the eventual maintenance of VRT effects over time.

## 5. Conclusions

The results obtained in our study on the effectiveness of a non-immersive VRT on re-learning FLS seem to be encouraging, especially for the spontaneous generalization to the natural environments of the re-learned skills. Specific trainings seem to be needed to promote improvements in such a domain, essential for the well-being and self-esteem in people with dementia.

The advantages of teaching by means of VR range from the possibility to learn in a safe and controlled environment, to enjoyably learn in an independent way, at any time, without the need of a constant presence of a trainer; furthermore, VR allows to completely and accurately collect data. The virtual modality we used is easily transferable to the patient home, where they might continue the rehabilitation with a remote control.

There are still some open questions: what is the VRT format that works best for different clinical groups? Is a fully immersive VRT better than a moderate or non-immersive VRT for people with dementia? Which devices are preferred by patients? How should task difficulties be adapted to the individual characteristics of patients? Future studies should try to provide answers to these questions. The future development of a technological system for VRT both at hospital and patient home is warranted. Additional versions of the apps are also needed, for their use on tablets (at home), as well as on virtual camera and “Oculus” (at the hospital), in order to provide a more immersive experience to patients and to collect data on the device preference of clinical groups of patients. Furthermore, a larger number of FLS apps is needed and planned.

## Figures and Tables

**Table 1:** Comparison between the clinical and demographic features of experimental and control groups at T1.

Feature	Experimental Group (*n* = 24)	Control Group (*n* = 18)	*p*=
**M-NCD etiology, *n***			
Vascular	11	4	0.41 ^a^
Alzheimer	8	7	
Fronto-temporal	2	3	
Other	3	4	
**M-NCD severity level, *n***			
MMSE < 18	6	6	0.54 ^a^
MMSE 18–23	16	10	
MMSE > 23	2	2	
**Gender, M/F, *n***	13/11	6/12	0.18 ^a^
Chronological age, years *	68.5 (61.8/72.1)	64.0 (61.3/69.8)	0.28 ^b^
**Education**, years *	8.0 (5.0/11.0)	8.0 (5.0/13.0)	0.47 ^b^
Mini Mental State Examination ^c^ *	20.1 (18.0/21.9)	19.6 (15.6/20.5)	0.19 ^b^
**Coloured Progressive Matrices**^c^ *	20.1 (18.1/24.3)	19.0 (14.4/24.3)	0.47^b^
**Visuo-spatial span (Corsi’s test)**^c^ *	4.3 (3.4/4.7)	3.6 (2.6/4.1)	0.19 ^b^
**Digit Span**^c^ *	4.5 (3.5/4.9)	4.1 (3.9/4.7)	0.35 ^b^
**Rey’s 15 words, Immediate Recall**^c^ *	26.0 (22.3/28.7)	21.3 (17.2/25.5)	0.1 ^b^
**Rey’s 15 words, Delayed Recall**^c^ *	3.5 (2.5/5.4)	2.0 (0.0/4.2)	0.55 ^b^
Frontal Assessment Battery *	9.5 (8.0/11.0)	8.5 (6.0/11.0)	0.75 ^b^
ADL *	6.0 (4.0/6.0)	6.0 (5.0/6.0)	0.62 ^b^
IADL, % *	37.5 (25.0/62.5)	62.3 (50.0/62.5)	0.3 ^b^
Information, correct responses *	24.0 (21.0/26.3)	24.0. (22.0/26.0)	0.99 ^b^
Information, total time, s *	264.5 (233.8/286.5)	314 (245.5/367.3)	0.04
Suitcase, correct responses *	5.0 (3.0/7.3)	5.0 (3.0/9.0)	0.5 ^b^
Suitcase, total time, s *	200.0 (128.0/263.8)	214.0 (173.0/395.0)	0.38 ^b^
Medicines, correct responses *	6.5 (4.3/8.0)	5 (3.5/6.5)	0.31 ^b^
Medicines, total time, s *	179.5 (156.3/230.5)	261.5 (218.8/377.3)	0.01
Supermarket, correct responses *	10.0 (7.0/13.0)	9.0 (5.0/12.0)	0.56 ^b^
Supermarket, total time, s *	270.0 (227.8/393.0)	343.0 (284.5/370.5)	0.52 ^b^

* Data reported as median (25–75th centile). M-NCD = major neurocognitive disorder; ^a^ Chi square test; ^b^ Mann Whitney’s U test; ^c^ corrected scores (age/education).

**Table 2:** Differences between the second (T3) and the first (T1) in vivo test in the experimental and control group, and results of the comparisons between the two groups.

Feature	Experimental Group (*n* = 24)	Control Group (*n* = 18)	z	Mann Whitney U Test, *p*=	Effect Size r *
Information, correct responses	3.0 (1.0/3.2) **	0.0 (−1.0/1.0)	2.78	0.005	0.43
Information, total time, s	−56.0 (−81.0/−23.2) **	−54.5 (−72.0/−12.0)	0.29	0.77	
Suitcase, correct responses	2.5 (1.0/4.0) **	0.0 (−1.3/0.0)	4.31	0.00001	0.66
Suitcase, total time, s	37.5 (−21.0/138.5)	−29.0 (−108.5/−14.5)	2.27	0.02	0.35
Medicines, correct responses	2.0 (1.0/3.0) **	0.0 (0.0/0.5)	3.32	0.00086	0.51
Medicines, total time, s	−36.0 (−85.5/−10.2) **	−7.0 (−73.2/34.2)	1.05	0.29	
Supermarket, correct responses	2.5 (1.0/5.0) **	0.0 (−0.5/1.0)	2.77	0.0056	0.43
Supermarket, total time, s	20.5 (−76.7/74.0)	−42.0 (−63.5/1.0)	0.91	0.36	

All values are shown as median (25th–75th centile). * r = z/√N; the r value of 0.1, 0.3 and 0.5 indicated a small, medium, and large effect size, respectively; ** within-group statistically significant difference at *p* ≤ 0.003 (Wilcoxon and Bonferroni correction).

**Table 3:** Results obtained by the experimental group during the VRT sessions at T2 (Friedman test for repeated measures).

VT Sessions	Correct Responses	Errors	Missing Responses	Clues	Execution Time
Information	0.2, =0.0002	NS	0.27, <0.00001	0.2, =0.0002	0.48, <0.00001
Suitcase	0.225, =0.00009	NS	0.3, <0.00001	0.226, =0.00008	0.45, <0.00001
Medicines	0.14, =0.03	NS	0.33, <0.00001	0.14, =0.036	0.44, <0.00001
Supermarket	0.33, <0.00001	NS	0.44, <0.00001	0.29, <0.00001	0.56, <0.00001

All values are shown as Kendall’s W coefficient of concordance, *p* value. Kendall’s W coefficient of concordance uses the Cohen’s interpretation guidelines of 0.1 (small effect), 0.3 (moderate effect), and above 0.5 as a strong effect.

## Data Availability

The data presented in this study are openly available in Mendeley at 10.17632/3kvmkrpm84.1.

## Supplementary Materials

The following are available online at https://www.mdpi.com/article/10.3390/s21175751/s1.
