# Supplementary material for: Functional Living Skills: A Non-Immersive Virtual Reality Training for Individuals with Major Neurocognitive Disorders
Source: Sensors (Basel). 2021 Aug 26;21(17):5751. doi: 10.3390/s21175751 (PMC8433763; doi:10.3390/s21175751)
Supplement: Supplementary file 1 [file sensors-21-05751-s001.zip › sensors-1286082-supplementary.pdf]

Description of apps in details

Title of apps	Initial Instruction (vocal and written)	Optional video-demonstration	App Content and Scenes	Max latency time for each action / response	Number of subsequent actions / responses	Number of clues for each incorrect responses	Clues description	Type of clues	Type of reinforcement of each correct response	Schedule of reinforcement
**Information**	"Now you will be asked some questions, please click on the button corresponding to the answer you think is correct. Good job!"	NO	Number of scenes: 1<br><br>30 questions appearing on the screen, one at a time, in verbal and written form, with multiple-choice answers; questions focus on general knowledge, personal, family, spatial and temporal orientation.	15 sec	30	1	The correct response is highlighted intermittently	Visual clue	Vocal positive rewards, such as "well done", "congratulation", "you are working carefully".<br>At the end of the task a general reward ("Well done! You worked careffully") along with applause is provided.	Variable ratio: VR2 or VR3 (reinforcement were provided in a variable way every two or three responses on average.
**Medicines**	"On the table there are some medicines and a reminder showing the times in which they must be taken. Now you will be given a time and you will touch the right medicine to take".	YES	Number of scenes: 1<br><br>Five medicine boxes are placed on a kitchen table, verbal instructions and a visual reminder explain when each drug should be taken; the patient is required to respond to 10 verbal requests, randomly presented during each session. The visual reminder is always available on the screen.	15 sec	10	1	The correct response is highlighted intermittently and vocally suggested	Vocal and visual clues	Vocal positive rewards, such as "well done", "congratulation", "you are working carefully".<br>At the end of the task a general reward ("Well done! You worked careffully") along with applause is provided.	Variable ratio: VR2 or VR3
**Suitcase**	"You have to	YES	Number of scenes: 1	15 sec	11	3, least to	1: a voice tells the	1:	Vocal positive	Variable ratio:

	go out for a weekend. Prepare a suitcase with a complete change o ìf clothes, including the hygienic bag. When you are done, close the suitcase”		Clothes to be placed in a suitcase are shown in some shelves. Patients are requested to perform dragging actions to put clothes in the suitcase.			most	patient what to do 2: the correct response is brighten for 3 sec 3: the computer performs the action and goes to the next step	vocal clue 2: vocal and visual clue 3: visual	rewards, such as “well done”, “congratulation”, “you are working carefully”. At the end of the task a general reward (“Well done! You worked careffully”) along with applause is provided.	VR2 or VR3
**Supermarket**	“You have to go shopping. Read the shopping list aloud, and click on each product in the list. Then, take the money to buy the 5 products, and put the list and the money in your wallet. Finally, go to the supermarket” .	YES	Number of scenes: 3<br><br>The first scene is a kitchen, with a shopping list (wich varies every day), money and wallet; patient is required to take money, put it in the walley, and to take the shopping list. He / she can control the shopping list at any time during the task. In the second scene patient has to take the listed products from a supermarket shelf and then put them in a shopping cart. In the third scene the patient has to pay for products at the cash counter. Patient is requested to perform dragging actions during the task.	15 sec	17	3, least to most	1: a voice tells the patient what to do 2: the correct response is brighten for 3 sec 3: the computer performs the action and goes to the next step	1: vocal clue 2: vocal and visual clue 3: visual	Vocal positive rewards, such as “well done”, “congratulation”, “you are working carefully”. At the end of the task a general reward (“Well done! You worked careffully”) along with applause is provided.	Variable ratio: VR2 or VR3

Che lavoro svolgi o svolgevi?

Operaio	Elettricista	Psicologo
Disoccupato	Regista	Ingegnere

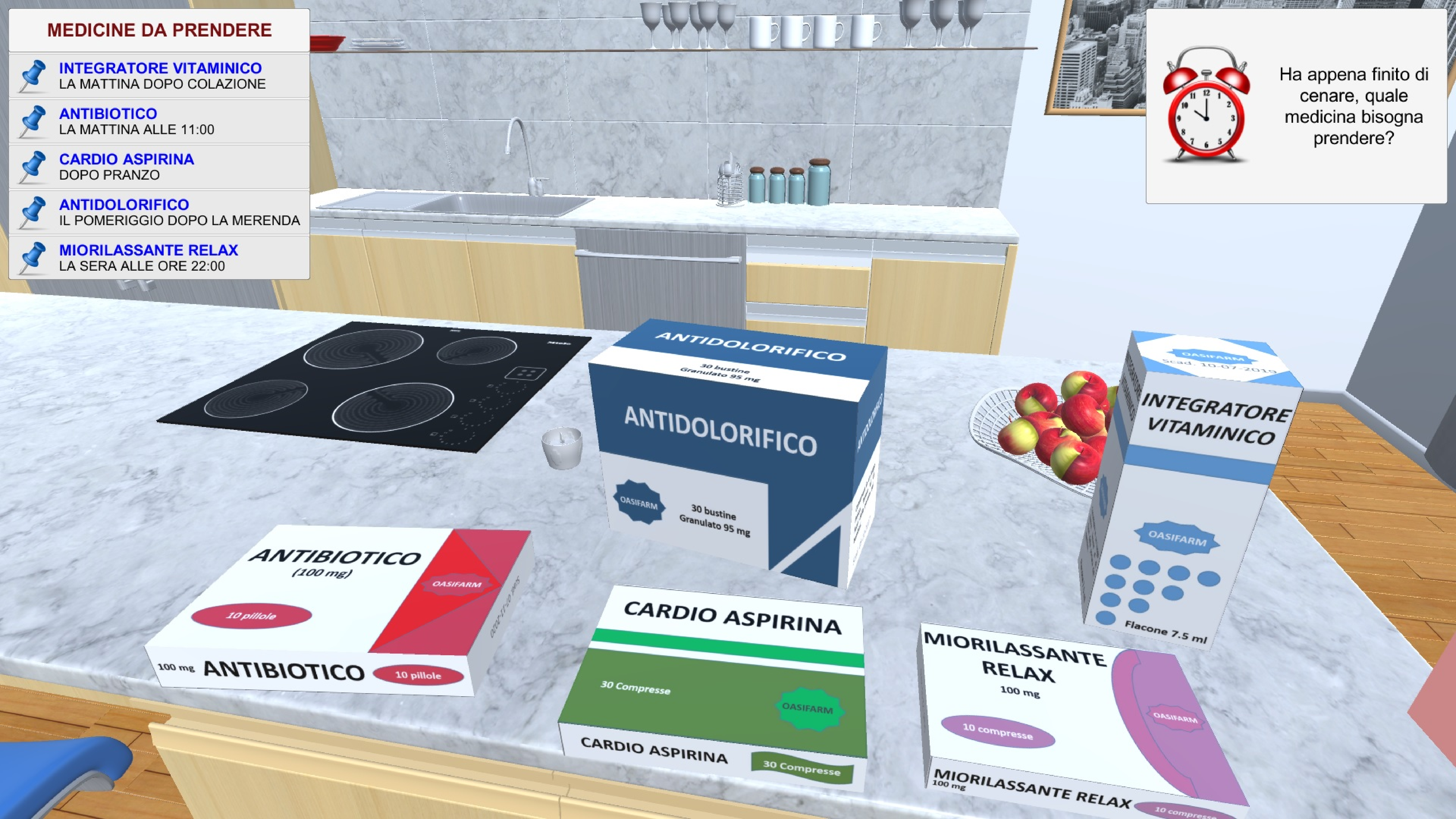
MEDICINE DA PRENDERE
INTEGRATORE VITAMINICO
LA MATTINA DOPO COLAZIONE
ANTIBIOTICO
LA MATTINA ALLE 11:00
CARDIO ASPIRINA
DOPO PRANZO
ANTIDOLORIFICO
IL POMERIGGIO DOPO LA MERENDA
MIORILASSANTE RELAX
LA SERA ALLE ORE 22:00
Ha appena finito di cenare, quale medicina bisogna prendere?
ANTIDOLORIFICO
30 bustine Granulato 95 mg
ANTIDOLORIFICO
OASIFARM
30 bustine Granulato 95 mg
INTEGRATORE VITAMINICO
OASIFARM
Flacone 7.5 ml
ANTIBIOTICO
(100 mg)
10 pillole
OASIFARM
100 mg ANTIBIOTICO 10 pillole
CARDIO ASPIRINA
30 Compresse
OASIFARM
CARDIO ASPIRINA 30 Compresse
MIORILASSANTE RELAX
100 mg
10 compresse
OASIFARM
MIORILASSANTE RELAX
100 mg

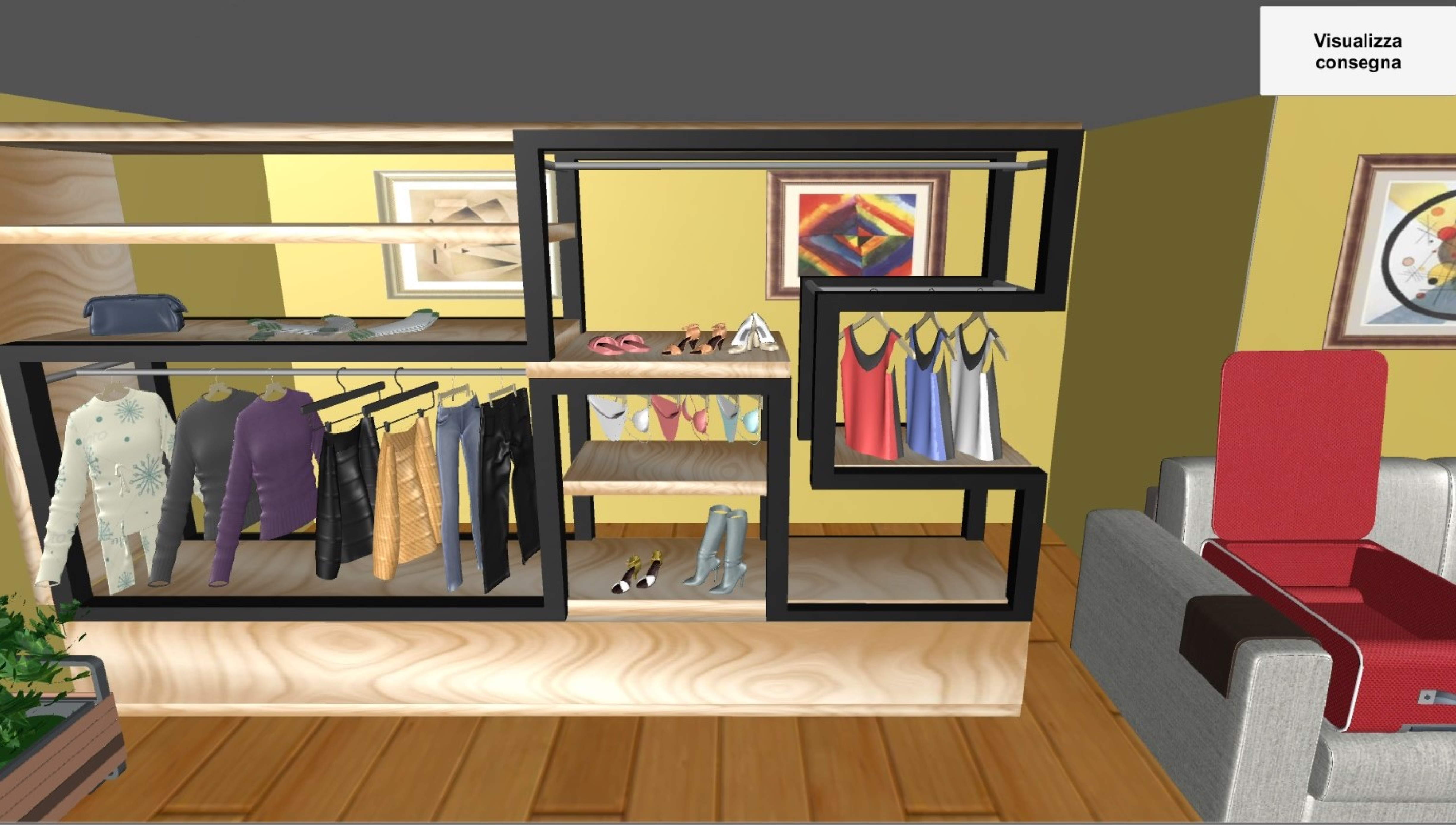
Visualizza
consegna

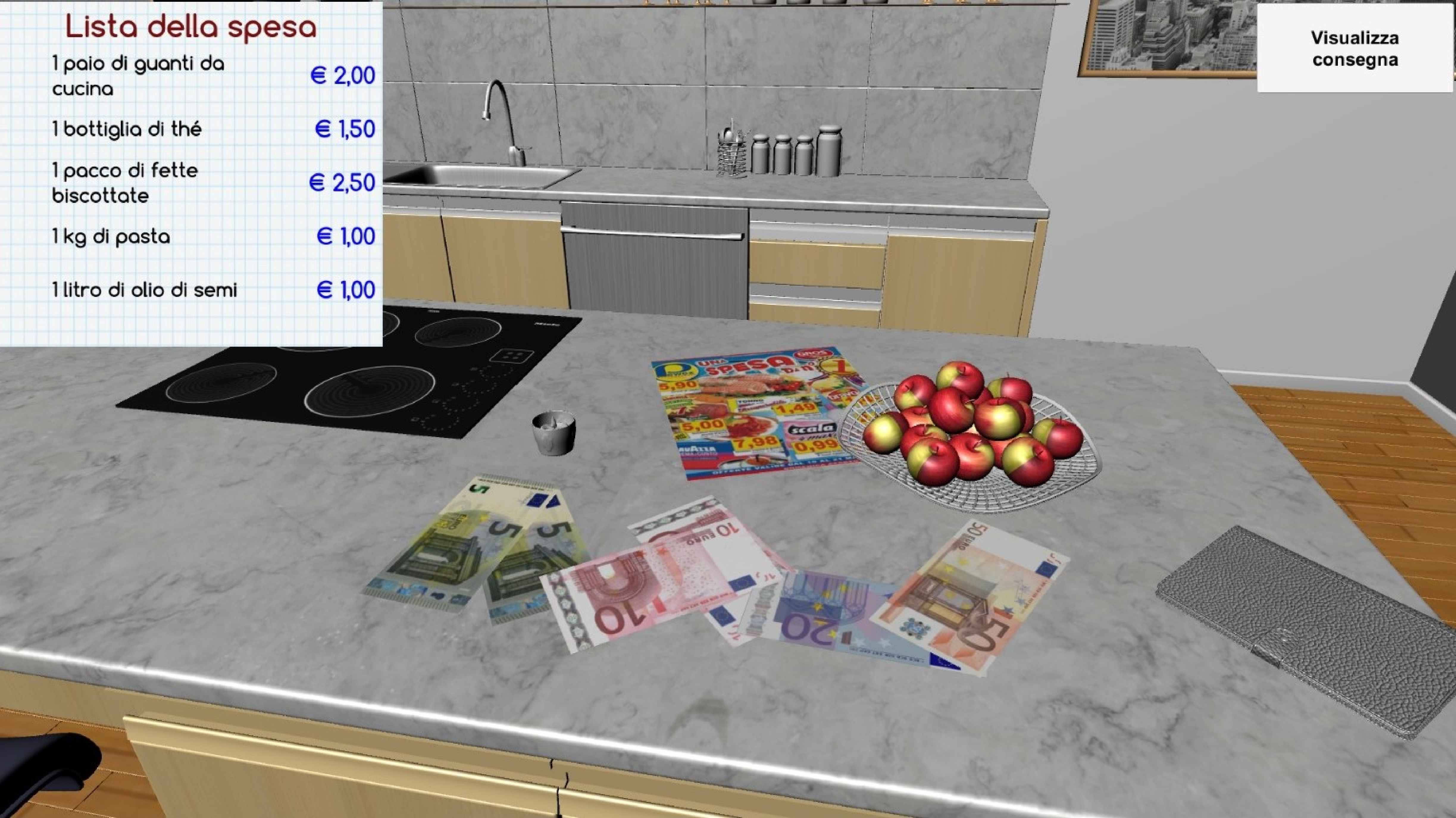
Lista della spesa
1 paio di guanti da cucina € 2,00
1 bottiglia di thé € 1,50
1 pacco di fette biscottate € 2,50
1 kg di pasta € 1,00
1 litro di olio di semi € 1,00
Visualizza consegna

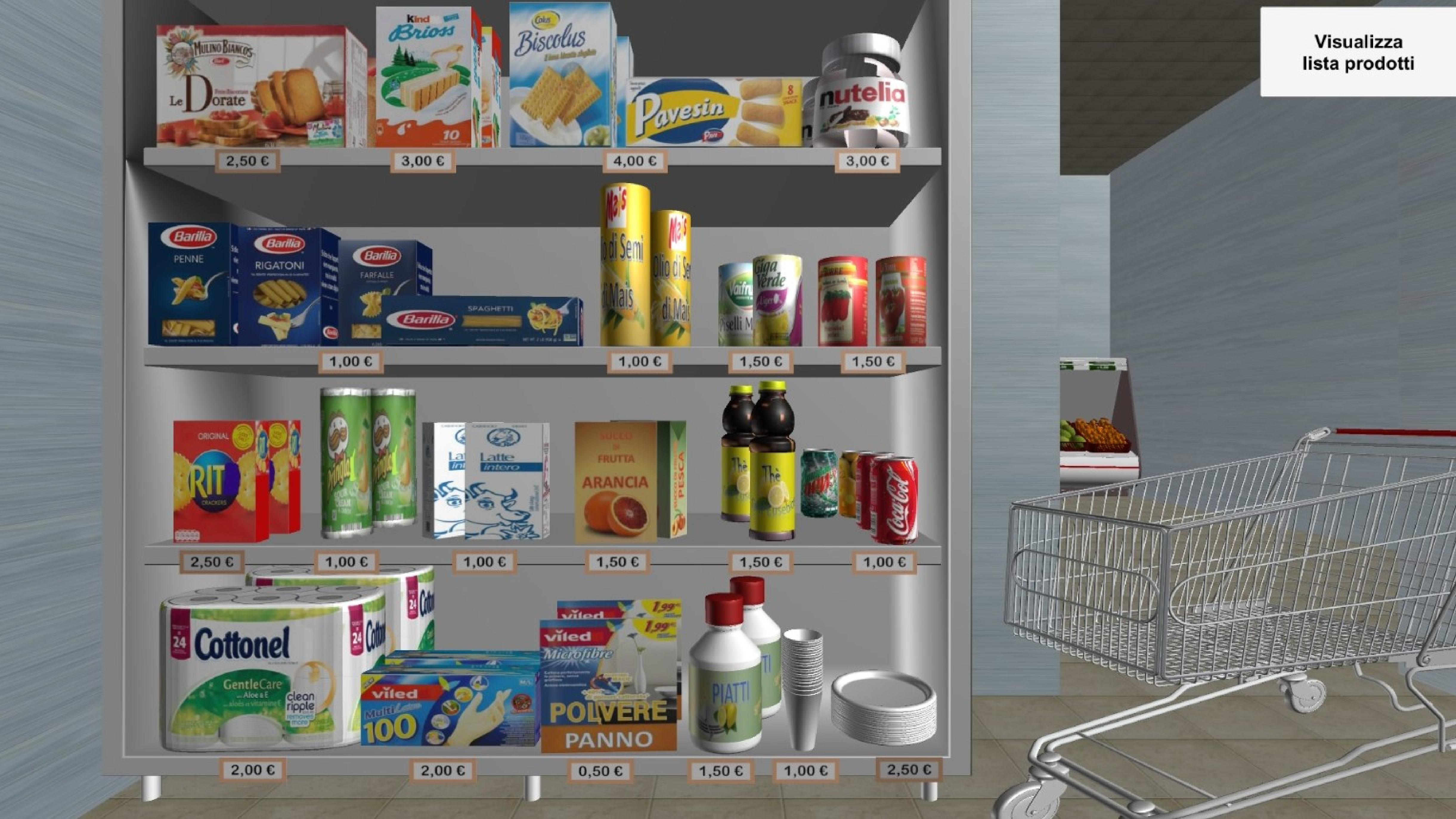

Visualizza lista prodotti
Mulino Bianco
Le Dorate
2,50 €
Kinder Brioss
3,00 €
Biscolus
Pavesini
4,00 €
nutella
3,00 €
Barilla PENNE
Barilla RIGATONI
Barilla FARFALLE
Barilla SPAGHETTI
1,00 €
Mais Olio di Semi di Mais
1,00 €
Giga Verde
1,50 €
1,50 €
ORIGINAL RITZ CRACKERS
2,50 €
Pringles
1,00 €
Latte intero
1,00 €
SUCCO DI FRUTTA ARANCIA
PESCA
1,50 €
Thè
1,50 €
Coca-Cola
1,00 €
Cottonel 24 GentleCare
2,00 €
vileda Multi 100
2,00 €
vileda Microfibre POLVERE PANNO 1,99
0,50 €
PIATTI
1,50 €
1,00 €
2,50 €

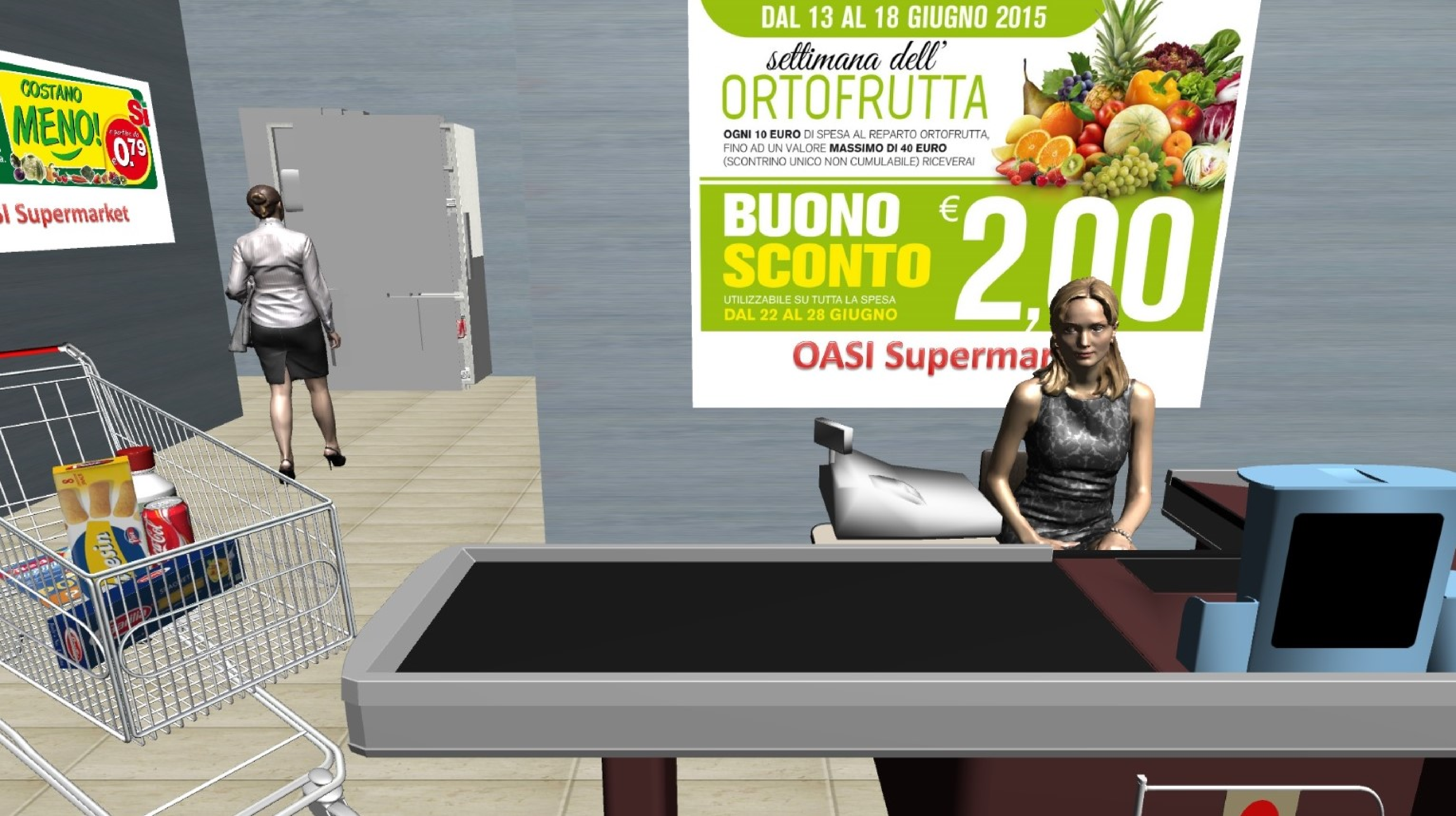
COSTANO
MENO!
0,79
SI Supermarket
DAL 13 AL 18 GIUGNO 2015
settimana dell'
ORTOFRUTTA
OGNI 10 EURO DI SPESA AL REPARTO ORTOFRUTTA, FINO AD UN VALORE MASSIMO DI 40 EURO (SCONTRINO UNICO NON CUMULABILE) RICEVERAI
BUONO SCONTO
€ 2,00
UTILIZZABILE SU TUTTA LA SPESA
DAL 22 AL 28 GIUGNO
OASI Supermar

ID	EG 1 - CG 2	VT sessions	etiology: Alzheimer=1; other degenerative=2; vascular =3: other=4	Years of Education	Chron. age	Male 1 Female 2	pre MMSE	post MMSE	pre CPM	post CPM	pre Corsi span	post Corsi span	pre digit span	post digit span	pre 15 rey words: immediate recall	post 15 rey words: immediate recall	pre 15 rey words: delayed recall	post 15 rey words: delayed recall	pre FAB scores	post FAB scores	pre ADL scores	post ADL scores	pre IADL: % of maintained abilities	post IADL: % of maintained abilities	number of devices used	satisfaction questionnaire positive items (MAX scores=16)	satisfaction questionnaire negative items (MAX scores=12)
1	1	10	2	5	59	2	16.97	21.97	24.5	25.5	4.35	4.35	4.29	4.29	22.4	28.4	6.2	7.2	6	10	6	6	75	75	2	12	1
2	1	12	4	3	78	2	19.5	25.24	19.5	28.5	3.02	4.02	4.96	3.96	33.2	37	6.8	9.1	6	10	4	4	62.5	62.5	1	13	0
3	1	20	3	8	54	2	25.7	29.97	32.5	33.5	4.35	4.35	4.04	4.04	40.07	41.07	5.02	8.02	10	15	5	5	62.5	62.5	2	14	0
4	1	12	3	5	62	1	16.27	21.27	30.5	30.5	3.56	2.56	4.29	4.29	17.1	19.1	3.8	3.8	8	10	6	6	37.5	37.5	1	12	1
5	1	10	3	5	69	2	19.27	25.27	22	25	4.36	4.36	4.51	4.51	41	44	9.4	7.4	8	11	4	4	37.5	37.5	1	10	0
6	1	16	3	8	68	1	23.53	26.53	17.5	22.5	3.44	3.44	5.35	6.35	22.9	39.9	1.9	3.9	10.7	11.7	5	5	37.5	37.5	1	10	0
7	1	15	3	5	65	1	20.27	25.27			4.25	4.25	3.39	3.39		41.1		6.8					62.5	62.5	1	12	0
8	1	20	2	8	62	2	20.53	23.53	20.5	19.5	5.5	5.5	4.35	4.35	28	27	5.5	5.3	8	8	4	4	62.5	62.5	1	10	0
9	1	20	1	8	71	1	17.2	18.2	26.5	27.5	5.36	5.36	4.49	4.49	28.9	31.9	3.6	2.6	8	11	6	6	12.5	12.5	4	10	0
10	1	20	3	8	68	1	20.53	24.53	26.5	30.5			3.35	2.35	30.9	49.9	5.9	8.9	8	9	2	2	12.5	12.5	2	13	0
11	1	13	3	3	59	1	18.24	20.2	18	18	4.69	3.69	3.43	4.43							3	3	25	25	1	14	0
12	1	12	4	5	74	1	24.03	26.03	31	32	4.69	4.69	4.65	4.65	28	34	0	5.1	11	9	3	3	25	25	1	5	1
13	1	17	3	5	71	2	19.9	21.03	22	17.4	4.66	4.66	4.65	4.65	40	43	8.4	6.4	6	11	6	6	25	25	1	8	1
14	1	20	1	8	80	1	22.2	28.2	23.5	18.5	3	4	5.65	6.65	22.1	34.1	3.3	6.3	16	15	5	5	37.5	37.5	1	8	0
15	1	20	1	13	61	1	19.49	24.49	17.5	19.5	5.85	4.85	5.02	5.02	12.3	19.3	1.6	2.6	9	10	6	6	25	25	4	13	1
16	1	20	1	5	77	1	22.03	23.03	24	24	3.25	3.25	6.82	5.82	20	21	3.1	3.1	10	9	6	6	37.5	37.5	1	10	1
17	1	20	1	8	64	1	18.53	17.53	15.5	13.5	3.56	3.56	5.23	5.23	27	27	4.3	3.3	12	12	6	6	25	25	1	14	0
18	1	20	1	5	70	2	14.03	16.03	15	22	4.66	4.66	3.51	4.51	27	32	2.4	3.4	7	8	6	6	50	50	1	2	1
19	1	20	1	8	81	2	21.2	20.2	22.5		2.57	3.57	4.87	4.87	27.1	24.1	3.3	3.3	15	8	6	6	62.5	62.5	1	7	0
20	1	15	3	17	50	2	20.31	20.31	16.31	22	3.41	5.41	2.6	3.6	22.7	34.7	0	0	13	13	3	3	12.5	12.5	4	16	0
21	1	15	3	13	81	2	12.86	23.86	19.5	29.5	3.15	3.15	4.44	5.44	17.4	25.4	2.6	2.6			6	6	75	75	2	8	0
22	1	20	4	8	55	1	21.97	24.97	20.4	25.4	4.51	4.51	3.13	3.13	22.3	21.3	0	0	11	15	6	6	12.5	12.5	2	16	0
23	1	20	1	18	82	1	21.85	23.85	20.8	27.8	3.69	3.69	2.36	2.36	23.6	37.6	5.2	7.2	10	15	6	6	50	50	3	2	2
24	1	20	3	5	74	2	17.03	19.03	18.2	24.2	2.15		4.65	4.65	25	32	3.1	5.1			6	6	87.5	87.5	2	12	0
A	2	0	2	3	70	2	24.24	23.24	13.5	19.5	0.72	0.72	5.79	5.79	31	48	7.40	9.40	5	6	6	6	62.5	62			
B	2	0	2	5	65	2	14.27	15.27	18.9	19.9	3.44	3.44	2.39	2.39	6.1	13.1	0.00	0.00	4	4	6	6	62.5	37.5			
C	2	0	4	13	63	1	23.49	27.49	29.3	34.5	5	4	5.02	6.02	25.03	31.03	1.06	3.06	8	13	6	6	50	66			
D	2	0	4	8	57	1	20.97	22.97	25.5	28.5	4.15	4.15	4.13	5.13	35.07	44.07	6.02	8.02	13	15	6	6	62.5	83			
E	2	0	1	13	69	1	20.49	20.49	19	16	4.11	3.11	4.13	6.13	26.1	23.1	4.20	4.20	14	12	6	6	62.5	80			
F	2	0	4	18	61	2	20.46		25		3.83				13.8		0.00		8		6	6	87.5	87.5			
G	2	0	3	5	67	2	20.27	24.27	20.5	28.5	2.56	4.56	4.51	4.51	25.1	29.1	4.80	4.80	13	13	5	5	62.5	62.5			
H	2	0	1	8	80	2	20.2	23.2	19.5	23.5	3.66	4.66	4.65	4.65	31.1	29.1	3.30	3.30	10	12	6	6	62.5	62.5			
I	2	0	3	8	53	2	19.97	26.97	30.5	34.5	4.07	4.07	4.04	5.04	19.07	25.07	2.02	8.02	10	12	6	6	62.5	83			
L	2	0	3	3	80	2	19.24	21.24	23.5	26.5	4.02	5.02	3.96	4.96	43.3	40.2	9.80	9.80	7	9	1	1	25	25			
M	2	0	1	12	72	2	18.86	21.86	18	27	4.11	4.11	5.12	4.12	17.1	22.1	0.00	5.20	6	8	5	5	25	25			
N	2	0	2	5	57	2	18.74	19.74	24.5	26.5	3.35	4.35	4.29	3.29	16.8	21.4	4.70	6.20	14	9	6	6	75	75			
O	2	0	1	13	56	2	15.99	20.99	8	17	2.9	2.9	3.92	2.92	16	13	1.50	1.50	11	13	4	4	50	50			
P	2	0	3	13	63	1	15.49	20.49	11	9	1	2	2.02	4.02	22.3	19.3	0.00	3.60	9	9	6	6	12.5	20			
Q	2	0	1	13	62	2	15.49	18.49	8.5	11.5	2	4	5.03	6.02	17.4	19.4	0.00	0.00	6	7	4	4	50	50			
R	2	0	1	5	65	2	12.27	22.27	17.5	19.5	2.44	2.44	3.39	4.39	20.1	29.1	3.80	5.80	6	12	5	5	50	50			
S	2	0	4	8	62	1	12.53	14.47	0	10	3.25	3.25	4.23	4.23	22.03	26.03	0.27	0.27	3	5	6	6	62.5	80			
T	2	0	1	5	72	2	22.03	28.03	17	33	3.69	4.69	3.51	4.51	20.5	27	2.04	3.04	11	13	5	5	50	50			

Feature	Experimental Group (n=24)	Control Group (n=18)	Mann-Whitney U test, p=
Information, correct responses at T1.	24.0 (21.0-26.3)	24.0 (22.0-26.0)	0.005
Information, correct responses at T3.	27.5 (23.8-28.0)	25.0 (23.0-26.0)	
Wilcoxon's matched pairs test, p=	0.00014	NS (0.66)	
Information, total time at T1, s	264.5 (232.8-286.5)	314.5 (245.5-367.3)	NS
Information, total time at T3, s	206.0 (174.0-227.8)	260.0 (231.5-299.5)	
Wilcoxon's matched pairs test, p=	0.00008	0.009	
Suitcase, correct responses at T1	5.0 (3.0-7.3)	5.0 (3.0-9.0)	0.00001
Suitcase, correct responses at T3	8.0 (5.8-10.0)	6.0 (3.0-7.0)	
Wilcoxon's matched pairs test, p=	0.00009	NS (0.17)	
Suitcase, total time at T1, s	200.0 (128.0-263.8)	214.0 (173.0-395.0)	0.02
Suitcase, total time at T3, s	209.5 (162.5-288.0)	200.0 (173.0-258.0)	
Wilcoxon's matched pairs test, p=	NS (0.43)	NS (0.09)	
Medicines, correct responses at T1	6.5 (4.3-8.0)	5.0 (3.5-6.5)	0.00086
Medicines, correct responses at T3	8.5 (7.3-10.0)	5.0 (2.0-8.0)	
Wilcoxon's matched pairs test, p=	0.0005	NS (0.87)	
Medicines, total time at T1, s	179.5 (156.3-230.5)	261.5 (218.8-377.3)	NS
Medicines, total time at T3, s	141.5 (105.3-168.5)	239.0 (202.5-317.5)	
Wilcoxon's matched pairs test, p=	0.001	NS (0,40)	
Supermarket, correct responses at T1	10.0 (7.0-13.0)	9.0 (5.0-12.0)	0.0056
Supermarket, correct responses at T3	13.0 (11.8-14.0)	9.5 (5.8-12.0)	
Wilcoxon's matched pairs test, p=	0.0007	NS (0.57)	
Supermarket, total time at T1, s	270.0 (227.8-393.0)	343.0 (284.5-370.5)	NS
Supermarket, total time at T3, s	296.5 (217.5-385.0)	247.0 (171.5-346.0)	
Wilcoxon's matched pairs test, p=	NS (0.69)	0.02	

Non immersive Virtual Reality Training on Functional Living Skill

SATISFACTION QUESTIONNAIRE

Now I'm going to ask you some questions about the IADL system. I kindly ask you to answer the questions truthfully. The response options are: LITTLE, ENOUGH or A LOT. Ask me for all the explanations you need if there is something that is not clear to you.

	**LITTLE**	**ENOUGH**	**A LOT**
1. Did you like completing the tasks with the FLS system?			
2. Did you feel comfortable while using the FLS system?			
3. Were FLS instructions easy to understand?			
4. Do you feel like you have learned new and interesting things?			
5. * Was there anything annoying about the FLS system?			
6. * Did you ever get tired while using the FLS system?			
7. * Did you ever get angry while using the FLS system?			
8. * Did you think the FLS system was not working properly?			
9. * Is there anything about the FLS system that you didn't like?			
10. Do you prefer using the FLS system rather than traditional rehabilitation in which you have to write down answers on the sheet?			
11. Would you like new games to be developed similar to the ones you used?			
12. Would you like to continue using the FLS system in the future?			
13. Would you recommend the use of the FLS system to other people?			

Score:

0 =little

1=enough

2 =a lot

Reverse scores in items marked with an asterisk.
